# Editorial: Functional Foods and Bioactive Compounds for Improving and Maintaining Digestive Health

**DOI:** 10.3389/fnut.2021.815370

**Published:** 2022-01-06

**Authors:** Amalia Yanni, Yiannis Kourkoutas

**Affiliations:** ^1^Laboratory of Chemistry, Biochemistry, Physical Chemistry of Foods, Department of Nutrition and Dietetics, Harokopio University, Athens, Greece; ^2^Laboratory of Applied Microbiology and Biotechnology, Department of Molecular Biology and Genetics, Democritus University of Thrace, Alexandroupolis, Greece

**Keywords:** functional foods, probiotics and prebiotics, digestive health, bioactive compounds, food safety

Nowadays, a marked, constantly increasing interest in developing functional foods aiming at the maintenance of digestive health, is witnessed. Foods with specific metabolic targets are at the spearhead of scientific research due to strong associations between the gut microbiome, oxidative stress, inflammation and metabolism (1).

Foods aiming at digestive health constitute the second-largest positioning platform behind products tailored to general wellbeing. Such food products usually contain probiotic microorganisms and/or prebiotic dietary bioactive fibers. According to the latest definition of FAO/WHO (2), probiotics are viable microorganisms (bacteria or yeasts) which, when administered in adequate amounts, confer a health benefit on the host, while prebiotics are non-digestible compounds that induce the growth or activity of beneficial microorganisms. To induce the health benefits, probiotic products need to contain an adequate number of live cells, able to proliferate and colonize in the gut (3). Ensuring the viability and functionality of probiotics and the effective modulation of microbiota through prebiotics, in order to provide the maximum health benefit to the host, is a complex and intriguing topic of investigation.

Beyond probiotics and prebiotics, other constituents, such as bioactive compounds with antioxidant and anti-inflammatory properties and compounds associated with a targeted metabolic response, belong to the spectrum of ingredients which are used in food development aiming at the enhancement of functional properties (4).

Currently, the functional market directed at digestive health is mainly focused on pharmaceutical products, such as pills or capsules, rather than processed foods (5). In this vein, this Research Topic highlights novel functional foods/ingredients with beneficial properties targeting improvement and maintenance of digestive health. In addition, current knowledge regarding the complex relationship between food composition with gut microbiota and digestive health and food safety aspects, which constitute an important factor determining the functional food market, are presented.

There is evidence that probiotics have a broad antitumor effect in colorectal cancer. However, the mechanism remains unclear. The effect of Bornlisy (BO)-cocktails of three probiotics on colitis-associated colon cancer (CAC) and the underlying mechanism were studied by Lu et al.. Treatment of CAC mice with BO resulted in decreased tumor loads and also inhibited proliferation and metastasis of CRC cells *in vitro*. The study suggested that BO may provide an intervention strategy for CRC therapy, while G-protein-coupled receptor 43 (GPR43) is a potential targeting receptor during BO treatment.

A soybean resistant protein (SPR)-containing diet, derived from kori-tofu, a traditional Japanese food, resulted in decreased serum level of lipopolysaccharide-binding protein and higher expression of Reg3γ, a chemical barrier, which plays an essential role in the segregation of the microbiota, thereby improving the intestinal barrier function in mice, as reported by Ogita et al.. In addition, SRP intake induced changes in the cecal microbiota, as observed by changes in β-diversity. SRP could be considered as a functional food component that may contribute to the maintenance of intestinal homeostasis, according to the researchers.

The role of hemicellulose-derived oligosaccharides (HDOs) as emerging prebiotics for the alleviation of diseases is discussed by Jana et al.. HDOs constitute functional ingredients to be incorporated in food products, since they play a significant role in the modulation of gut microbiota and serve as substrates for the probiotic production of health-promoting substances, such as short-chain fatty acids (SCFAs). Butyrate generated by the selective fermentation of oligosaccharides, along with other SCFAs reduce gut inflammation. The role of HDOs in the alleviation of autoimmune diseases like inflammatory bowel disease (IBD), and others, such as, obesity, diabetes and urinary tract infection, as well as in the antimicrobial resistance, through the modulation of the gut microbiota is presented, while the mechanism of oligosaccharide utilization and disease mitigation is also discussed.

Western diets have been associated with intestinal inflammation in contrast to plant-based diets, which often result in a reduced intake of total fats and meats and an increased intake of plant fibers that may contribute to reduced inflammation. A better understanding of the role of diet in intestinal inflammation may contribute to novel dietary interventions and to the use of specific foods or food supplements that can support the management of IBD. The influence of plant-based dietary components on the clinical disease course of IBD along with the benefits and possible limitations of plant-derived dietary components in the treatment of IBD are discussed in a review paper by Lauridsen et al..

Colonization ability of foodborne microbes and impact on human gut microbiota was discussed by Roselli et al. in a systematic review, which aims to provide the scientific literature addressing the connection between foodborne and gut microbiome and map gut microorganisms originating from fermented foods, either traditional or added with probiotics, among others. The review highlighted experimental approaches and study designs, which could be better standardized to improve comparative analysis of published datasets.

Apart from human health, an upsurge of interest is observed in food safety, due to the concerns associated with applying antibiotics in feed, resulting in drug resistance in pathogenic bacteria and drug residues in livestock products. Hence, the effect of dietary supplementation with mixed organic acids (MOA) on intestinal health, enzyme activity, and antioxidative characteristics in broilers was studied by Ma et al.. Dietary supplementation with MOA improved the health status of broilers, promoted the immune function, enhanced antioxidant enzymes, total antioxidant capacity and the expression of tight junction proteins and resulted in modulation of cecum microbiota.

Further research employing standardized and trans-disciplinary approaches is of pivotal importance for the understanding of gut microbiota and foods- probiotics- and prebiotics-interactions and their association with digestive health.

## Author Contributions

All authors listed have made a substantial, direct, and intellectual contribution to the work and approved it for publication.

## Conflict of Interest

The authors declare that the research was conducted in the absence of any commercial or financial relationships that could be construed as a potential conflict of interest.

## Publisher's Note

All claims expressed in this article are solely those of the authors and do not necessarily represent those of their affiliated organizations, or those of the publisher, the editors and the reviewers. Any product that may be evaluated in this article, or claim that may be made by its manufacturer, is not guaranteed or endorsed by the publisher.
